# Automated segmentation of trabecular and cortical bone from proton density weighted MRI of the knee

**DOI:** 10.1007/s11517-018-1936-7

**Published:** 2018-12-05

**Authors:** Hao Chen, André M. J. Sprengers, Yan Kang, Nico Verdonschot

**Affiliations:** 1 Department of Biomechanical Engineering, University of Twente, Drienerlolaan 5, 7500 AE Enschede, the Netherlands; 2 Orthopaedic Research Laboratory, Radboud University Medical Center, Geert Grooteplein-Zuid 10, 6525 GA Nijmegen, the Netherlands; 3 Sino-Dutch Biomedical and Information Engineering School, Northeastern University, No. 195 Chuangxin Road, Hunnan District, Shenyang, 110169 China

**Keywords:** Level set, MRI, Bone segmentation, Automatic, Bias correction

## Abstract

Patient-specific implant design and pre- and intra-operative planning is becoming increasingly important in the orthopaedic field. For clinical feasibility of these techniques, fast and accurate segmentation of bone structures from MRI is essential. However, manual segmentation is time intensive and subject to inter- and intra-observer variation. The challenge in developing automatic segmentation algorithms for MRI data mainly exists in the inhomogeneity problem and the low contrast among cortical bone and adjacent tissues. In this paper, we proposed a method for automatic segmentation of knee bone structures for MRI data with a 3D local intensity clustering-based level set and a novel approach to determine the cortical boundary utilizing the normal vector of the trabecular surface. Application to clinical imaging data shows that our method is robust to MRI inhomogeneity. In comparing our method to manual segmentation in 18 femurs and tibiae, we found a dice similarity coefficient (DSC) of 0.9611 ± 0.0052 for the femurs and 0.9591 ± 0.0173 for tibiae. The average surface distance error was 0.4649 ± 0.1430 mm for the femurs and 0.4712 ± 0.2113 mm for the tibiae. The results of the automatic technique thus strongly corresponded to the manual segmentation using less than 3% of the time and with virtually no workload.

**Graphical abstract:**
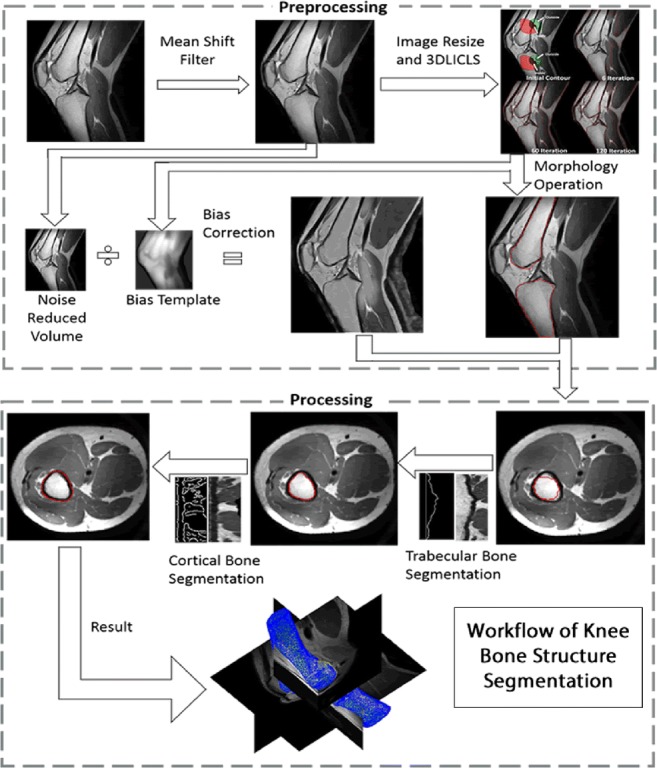
ᅟ

ᅟ

## Introduction

**Fig. 1:**
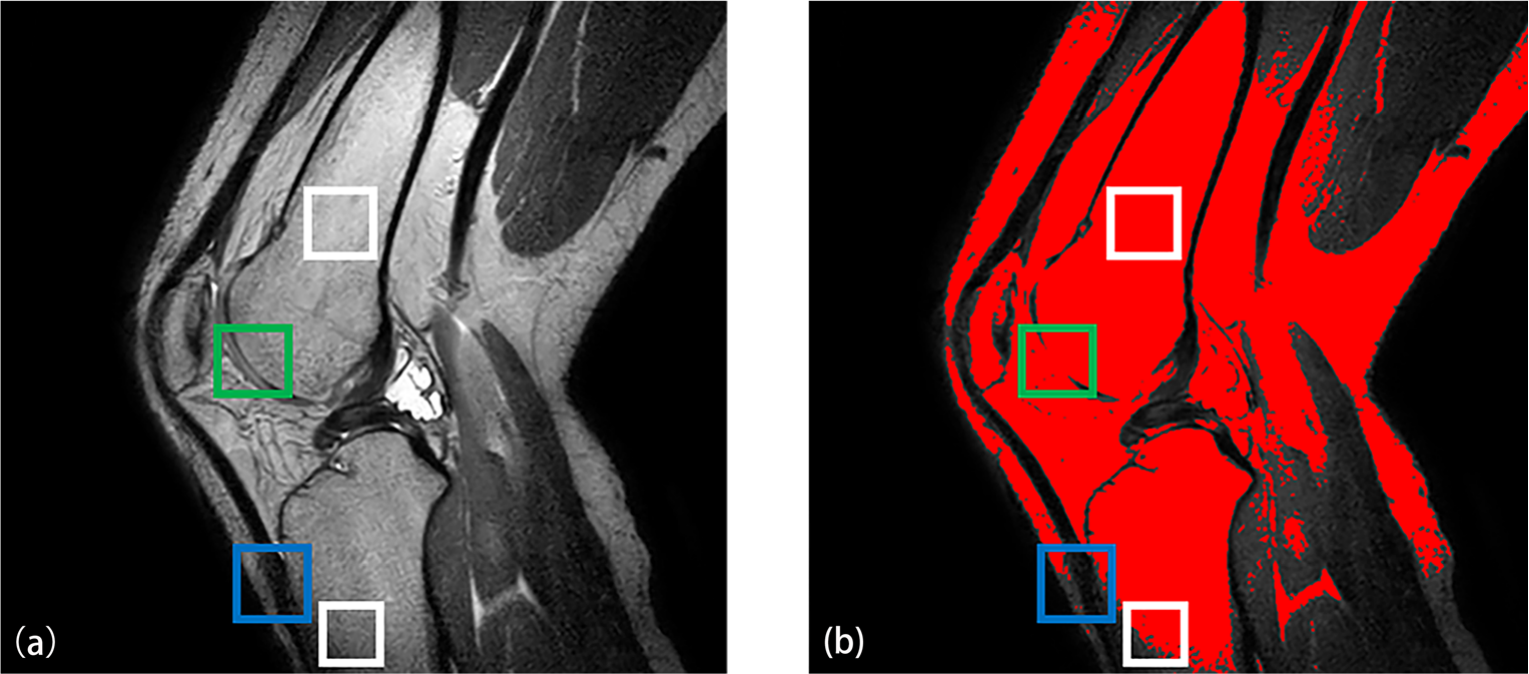
**a** Knee MRI image in proton density weighted (PDW) sequence. **b** Segmentation with Ostu’s method (red mask is the segmentation mask). (White box represents the inhomogeneity problem, green box represents the issue of low contrast between trabecular bone and infrapatellar fat and blue box represents the low contrast problem between cortical bone and ligament)

Fast and accurate segmentation of knee bone structures from MRI data is a topic of increasing interest as its applications continue to broaden from direct diagnostic purposes [20] to the creation of 3D finite element models [17], optimizing implant design [22] and pre- and intra-operative planning [18]. However, accurate automated segmentation is hampered by two problems:

1. Intensity inhomogeneity due to MRI inherent problems (coil sensitivity and B1 inhomogeneity) can cause a slow varying intensity gradient as can be noticed from the difference in brightness of trabecular fat in the two white boxes in Fig. 1)
2. Low contrast between the structures of interest (trabecular bone and infrapatellar fat (green box in Fig. 1), cortical bone and ligament (blue box in Fig. 1)).

Multiple approaches have been used for knee joint segmentation such as thresholding, region growing, deformable models, clustering methods and atlas-guided approaches [1]. In 2010, several automated segmentation methods were assessed in the grand challenge competition for segmenting cartilage and bone in knee MRI data [10]. Prior knowledge-based models, such as statistical shape models and atlas-based methods, seemed to outperform pixel-based methods [9]. These methods, however, require data set training and may be less suitable for pathologies that are not incorporated in the training data. Hence, an alternative method to segment the image without training may be challenging but is desirable from both clinical and research perspective.

To the best of our knowledge, Lorigo et al. were the first to apply active contours to segment MRI-based knee joint images [15] without utilization of training data. The texture information based on vector-valued geodesic snakes with local variance was incorporated into the active contour framework to detect the trabecular bone from other structures. This kind of method to detect regions of interest through evolving contours or surfaces under constraints from a given image has been largely accepted in the segmentation field [1, 14, 23, 24]. To include cortical bone, Pang et al. added two forces driven active contour model to segment knee structures with fat-suppressed MR sequences, which included the directional vector field convolution (DVFC) force and coupled prior shape model [16]. Furthermore, Dodin et al. proposed a ray casting technique to detect the femur and tibia boundary slice by slice in sagittal direction [5]. Shan et al. proposed a multi-atlas-based method to extract the femur and tibia mask [19].

Although many attempts were put into automating these segmentations, problems in MRI inhomogeneity and weak edges remain challenging, especially for an effective way to estimate the boundary between the cortical bone and adjacent tissue with similarly low intensity, i.e. ligament.

In this paper, we propose an automatic segmentation for trabecular and cortical bone of the femur and tibia in a clinically relevant MR sequence, proton density weighted contrast [21]. We use a local energy-based level set method to obtain a 3D rough segmentation of trabecular bone and correct the image data from the inhomogeneity problem. Subsequently, we generate intensity lines slice by slice based on the rough trabecular masks. Then, we optimize the trabecular boundary based on the intensity lines and propose an iterative process to detect the cortical boundary.

The remainder of this paper is organized as follows: In Section 2, we introduce the MRI data, the segmentation pipeline and related methods. In Section 3, we compare the results of manual segmentation and segmentation using our method on proton density weighted MRI data. Finally, Sections 4 and 5 summarize this research, reviews the results in comparison to other studies and discusses the future plans.

## Materials and methods

### MRI image acquisition

A proton density weighted (PDW) contrast MRI sequence was chosen in this study as it is frequently used to assess pathologies of the knee in a clinical setting [11, 21]. The PDW contrast provides data in which the ligaments, menisci and cartilage can be simultaneously assessed for diagnosis with a reasonable scanning time. In the PDW contrast, all relevant structures are displayed in different intensities ranging from high to low: fatty tissue (i.e. both infrapatellar fat and trabecular bone), cartilage, muscle, ligament and virtually no signal in the cortical bone. Scans were acquired with an eight-channel rigid coil in a 3.0-T Philips scanner. Further sequence details are as follows: FOV = 200 × 200 × 200 mm, voxel size = 0.35 × 0.35 × 0.52 mm for six of the data sets and 0.60 × 0.60 × 0.90 mm for the other 12, flip angle = 90, TR/TE = 1000/32.18 ms and scan duration = 6 min. All data was interpolated to 0.90 × 0.90 × 0.90 mm. To test the robustness of the proposed segmentation pipeline, a total of 18 data sets were used in this study. This study was approved by the local IRB and written informed consent was provided by all subjects prior to the study.

### Segmentation pipeline

**Fig. 2:**
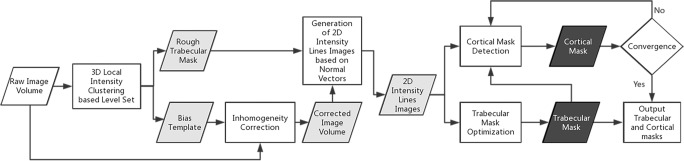
Segmentation pipeline for the proposed method. Rectangular boxes represent applied methods. White, grey and black parallelograms represent input data, intermediate data and output results, respectively

Figure 2 shows a schematic representation of the pipeline for the proposed automated method, which includes 3D local intensity clustering-based level set (3DLICLS), inhomogeneity correction, generation of 2D intensity line image along the normal vectors of the rough surface, trabecular mask optimization and cortical mask detection. Also, the required input data, intermediate data and corresponding output data are described in Fig. 2.

### Rough segmentation of the trabecular surface and inhomogeneity correction

In this section, we introduce a local energy-based level set method to both obtain a rough segmentation of the trabecular boundary and a bias field to remove any inhomogeneity from the data.

#### 3D local intensity clustering-based level set

In 2011, Li et al. proposed a local intensity clustering framework to segment the region of interest simultaneously with solving the inhomogeneity problem [12]. We extended this method to 3D in this study. Suppose the observed volume is *V*:

1 $$ V= bJ+{I}_{noise} $$

where *J* represents the actual 3D volume components; *b* is the 3D bias field, which accounts for the intensity inhomogeneity among the volumes and is slowly varying; and *I*_*noise*_ is the Gaussian noise with zero mean. We proposed to use the mean shift filter [4] to reduce the noise influence in this study, which leads to the model becoming *V* = *bJ*.

**Fig. 3:**
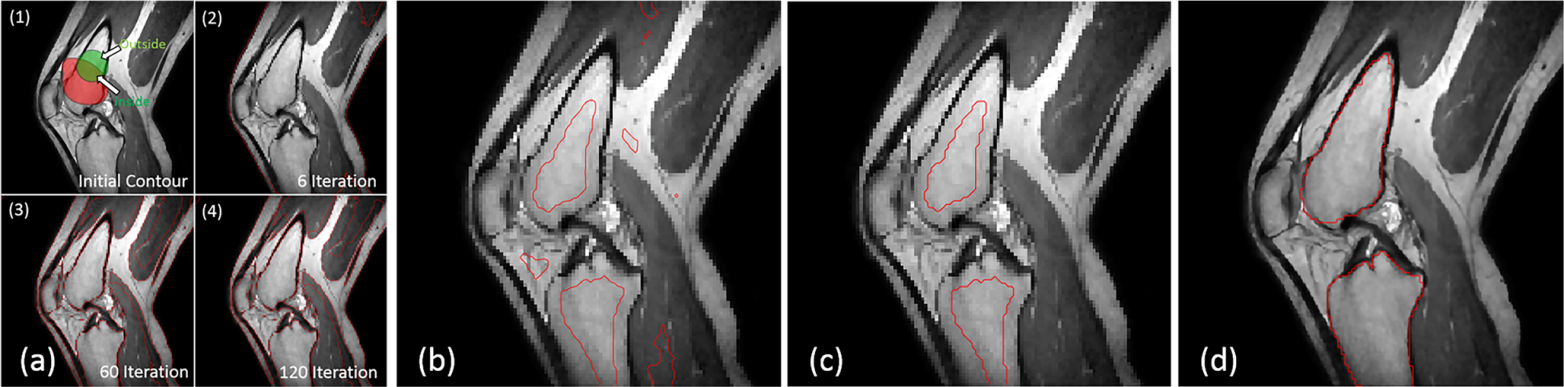
**a** Evolution of 3DLICLS: (1): red object is the initial contour example, green circle is used to calculate the inside energy (*e*_1_) and outside energy (*e*_2_); (2), (3), and (4): red contours are the boundary results in iterations 5, 14 and 60. **b** Red contour represents the boundary after erosion. **c** Red contour represents selected area for femur and tibia. **d** Red contour represents the dilated rough trabecular mask for femur and tibia

Based on the model, the essential ideas of 3DLICLS to segment interested object in image with intensity inhomogeneity are introducing a kernel function to define local energy function and introducing a bias variable to define the inhomogeneity template as follows:

2 $$ E(C)={\int}_{\varOmega }{\int}_{inside(C)}{K}_{\sigma}\left(x-y\right){\left|V(x)-b(y){c}_{inside}\right|}^2 dydx+{\int}_{\varOmega }{\int}_{outside(C)}{K}_{\sigma}\left(x-y\right){\left|V(x)-b(y){c}_{outside}\right|}^2 dydx $$

where *V* : *Ω* ∈ *R* is an input volume, *x*, *y* ∈ *Ω*, *K*_*σ*_ is a Gaussian kernel with standard deviation *σ*, *c*_inside_and *c*_outside_ represent the constant intensity inside and outside the contour *C* (such as dark green and light green in Fig. 3a (1)), respectively, and *b* is the inhomogeneity template. The reason for introducing the kernel function is to calculate the energy based on local information, while the reason for introducing the bias variable is to detect the target in the situation with inhomogeneity.

According to level set theory, contour, *C* ⊂ *Ω*, can be represented by the zero level set of a Lipschitz function *ϕ* : *Ω* ∈ *R* [3]. To minimize the cost function E with respect to *ϕ*, the gradient descent method is applied, $\frac{\partial \phi }{\partial t}=-\frac{\partial E}{\partial \phi }$, and thus, we can obtain the curve evolution equation as:

3 $$ \frac{\partial \phi }{\partial t}=-{\delta}_{\varepsilon}\left(\phi \right)\left({e}_1-{e}_2\right) $$

In order to stabilize the evolution of the level set function, a distance regularized term [13] is incorporated into (3). Furthermore, Euclidean length term is included to regularize the zero contour of *ϕ*. Finally, the final evolution equation is as follows:

4 $$ \frac{\partial \phi }{\partial t}=-{\delta}_{\varepsilon}\left(\phi \right)\left({e}_1-{e}_2\right)+v{\delta}_{\varepsilon}\left(\phi \right)\mathit{\operatorname{div}}\left(\frac{\nabla \phi }{\mid \nabla \phi \mid}\right)+\mu \left({\nabla}^2\phi -\mathit{\operatorname{div}}\left(\frac{\nabla \phi }{\mid \nabla \phi \mid}\right)\right) $$

In (4),

5 $$ \Big\{{\displaystyle \begin{array}{c}{e}_1(x)={\int}_{\varOmega }{K}_{\sigma}\left(y-x\right){\left|V(x)-b(y){c}_{inside}\right|}^2 dy\\ {}{e}_2(x)={\int}_{\varOmega }{K}_{\sigma}\left(y-x\right){\left|V(x)-b(y){c}_{outside}\right|}^2 dy\end{array}} $$

During the evolution, the representatives of constant and the bias template must be updated. Based on above assumption model, *V* could approximately be expressed as the multiplication of *b* and constant *c*, and thus, the updated form of *c*_*inside*_ and *c*_*outside*_ are as follows:

6 $$ \Big\{{\displaystyle \begin{array}{c}{c}_{inside}=\frac{\int \left({b}^{\ast }{K}_{\sigma}\right)V{H}_{\varepsilon}\left(\phi \right) dy}{\int \left({b}^{2\ast }{K}_{\sigma}\right){H}_{\varepsilon}\left(\phi \right) dy}\\ {}{c}_{outside}=\frac{\int \left({b}^{\ast }{K}_{\sigma}\right)V\left(1-{H}_{\varepsilon}\left(\phi \right)\right) dy}{\int \left({b}^{2\ast }{K}_{\sigma}\right)\left(1-{H}_{\varepsilon}\left(\phi \right)\right) dy}\end{array}} $$

and regarding *b*, the optimal bias filed, $\widehat{b}$, that minimized the energy *E* can be updated as follows:

7 $$ \widehat{b}=\frac{\left(V\left({c}_{inside}{H}_{\varepsilon}\left(\phi \right)+{c}_{outside}\left(1-{H}_{\varepsilon}\left(\phi \right)\right)\right)\right)\ast {K}_{\sigma }}{\left({c_{inside}}^2{H}_{\varepsilon}\left(\phi \right)+{c_{outside}}^2\left(1-{H}_{\varepsilon}\left(\phi \right)\right)\right)\ast {K}_{\sigma }} $$

Similar to previous level set-based method, such as [24], Heaviside function *H* and Dirac function *δ* used in above equation are as follows:

8 $$ \Big\{{\displaystyle \begin{array}{c}{H}_{\varepsilon }(x)=\frac{1}{2}\left[1+\frac{2}{\pi}\arctan \left(\frac{x}{\varepsilon}\right)\right]\\ {}{\delta}_{\varepsilon }(x)=\frac{1}{\pi}\cdot \frac{\varepsilon }{\varepsilon^2+{x}^2}\end{array}},x\in R $$

The selected iterations from the evolution are shown in Fig. 3a. Nevertheless, the output result of 3DLICLS includes not only the trabecular bone but also may include the infrapatellar fat, as Fig. 3a (4) shows. In order to obtain rough segmentations of the femur and tibia, a 3D spherical-shaped erosion kernel with radius of 5 mm was applied (Fig. 3b). After erosion, the femur and tibia bone area are separated from infrapatellar fat using a connectivity search (Fig. 3c). Then, an image dilation operation with the same kernel size of erosion finalizes the result, a rough segmentation of the trabecular bone of femur and tibia (see Fig. 3d). The basic theory of 3DLICLS also supports multi-phase detection [12]. In this study, we aimed to use it to position the trabecular bones roughly, and thus, the two phase model was selected.

#### Inhomogeneity correction

**Fig. 4:**
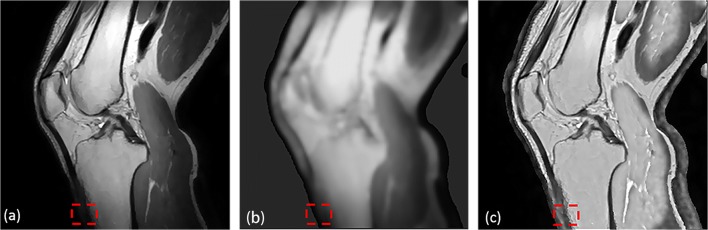
Inhomogeneity correction. **a** Original data. **b** Inhomogeneity template. **c** Inhomogeneity corrected data. (Red box represents the region can be optimized to segment)

The 3DLICLS process results in a rough segmentation of the trabecular bone of femur and tibia and a bias template of the complete FOV as shown in Fig. 4b. This bias template is used to remove inhomogeneity, and the bias-corrected volume is computed as:

9 $$ {V}_{corrected}=V/b $$

where *V*_*corrected*_ is the corrected volume, *V* is the original volume and *b* is the bias template from 3DLICLS. The corrected image slice is shown in Fig. 4c and the comparison between before and after correction is indicated by the red box in Fig. 4a, c**.**

### Generating normal vectors on the rough trabecular surface

**Fig. 5:**
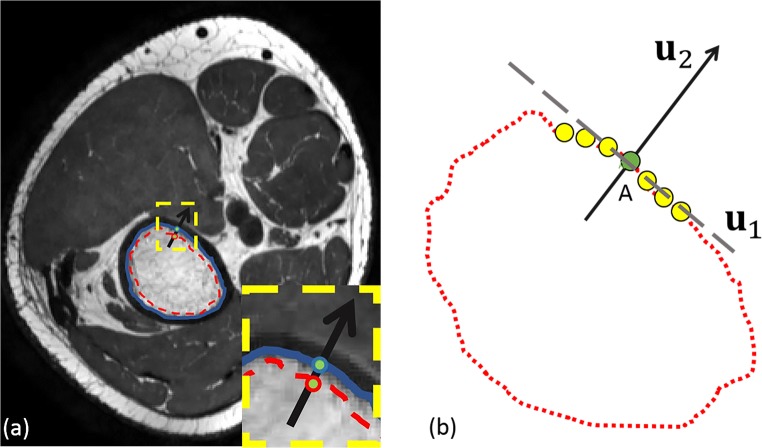
Illustration of normal vector calculation. **a** Red dash contour is the result of 3DLICLS, blue solid contour is target boundary and yellow dash rectangle is the enlarged area. **b** Black arrow is the normal vector, grey dash line is the tangent line of the contour and yellow circles are the neighbor points of target *A*. **u**_1_ and **u**_2_represent the tangent and normal vectors

Previous steps provide only the rough shape of the trabecular boundary. To obtain the precise trabecular bone boundary, an intensity line is generated along the normal vector of the trabecular surface (slice by slice), as Fig. 5a shows. To determine the normal vector of each point in the contour, such as green point A in Fig. 5b, we can apply singular value decomposition (SVD) among its neighbor points (yellow points) and itself (green point). In the case of point A, the coordinates of the points form the matrix *M*_*A*_,

10 $$ {M}_A=\left[\begin{array}{c}{x}_1\kern0.5em {x}_2\kern0.5em ...\kern0.5em {x}_n\\ {}\begin{array}{cccc}{y}_1& {y}_2& ...& {y}_n\end{array}\end{array}\right] $$

To obtain optimal solution in least squares sense, the first and second rows of *M*_*A*_ are corrected by their respective average *x*_*mean*_ and *y*_*mean*_, i.e. ${x}_n^{\hbox{'}}={x}_n-{x}_{mean}$, and obtain:

11 $$ {M}_A^{\hbox{'}}=\left[\begin{array}{c}{x}_1^{\hbox{'}}\kern0.5em {x}_2^{\hbox{'}}\kern0.5em ...\kern0.5em {x}_n^{\hbox{'}}\\ {}\begin{array}{cccc}{y}_1^{\hbox{'}}& {y}_2^{\hbox{'}}& ...& {y}_n^{\hbox{'}}\end{array}\end{array}\right] $$

Using SVD, ${M}_A^{\hbox{'}}$ is then decomposed into three parts, *U*, *Σ* and *V*,

12 $$ {M}_A^{\hbox{'}}= U\varSigma {V}^T $$

from which *U* provides the orthonormal vectors, **u**_1_ and **u**_2_. **u**_1_ is the tangent unit vector of point A, while **u**_2_ is the normal unit vector (the vector we use in this study). We refer to [2] for further explanation on *U*, *Σ* and *V*.

**Fig. 6:**
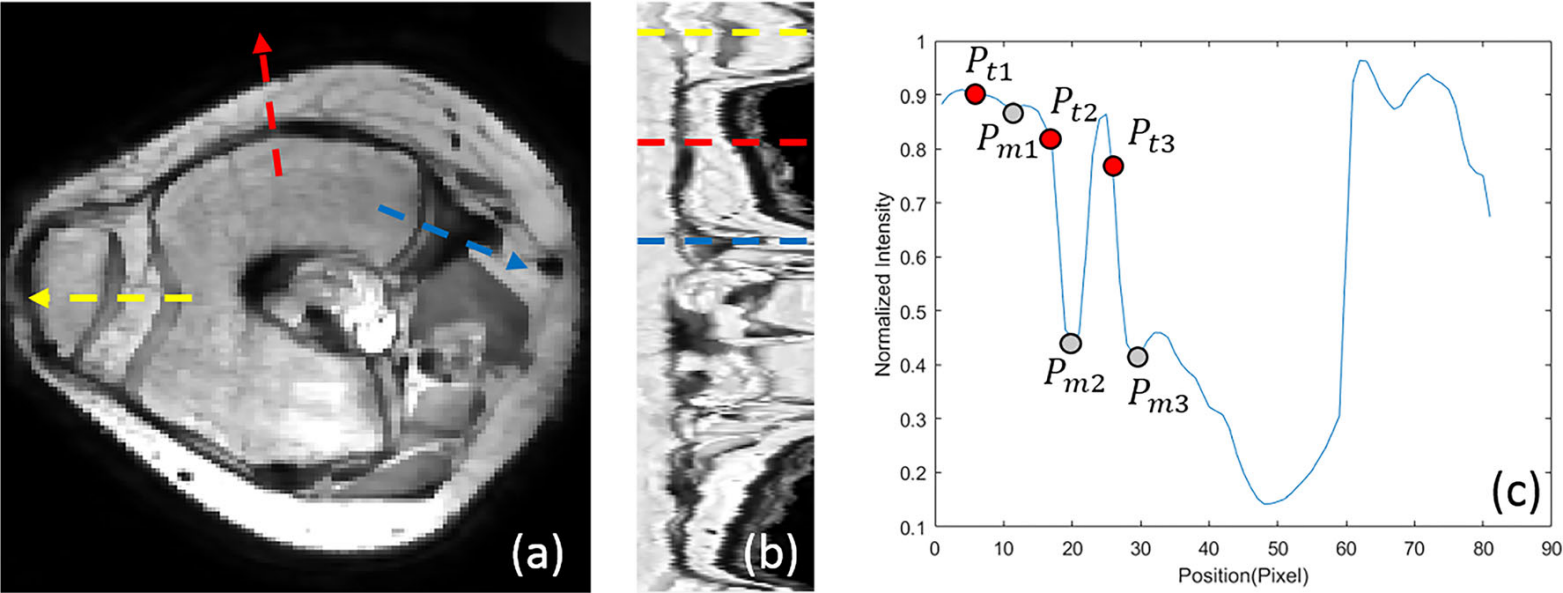
**a** Transverse view of femur. The arrow with different color corresponds to the different colored dash line in **b**. **b** 2D intensity line image with different colored dash line. **c** Intensity line of blue-dashed line on **b** (grey point represents the local minimum, red point represents trabecular candidate)

In a pilot study, a length of 45 mm for the intensity line along the normal vector (15 mm inward and 30 mm outward) was found to be adequate for robust inclusion of the precise trabecular boundary. Combining all intensity lines around the trabecular bone, an intensity line-based 2D image (IL2DI) is constructed, as Fig. 6b shows.

### Determination of the precise trabecular boundary

From the resulting 2D intensity lines, we now determine the precise trabecular surface slice by slice. Figure 6 shows a transverse slice of the femur (a), the complete set of IL2DI (b) and a typical intensity line (c). For each intensity line, trabecular candidate points *P*_*t*_ are defined as the point of maximum decline before a local minimum *P*_*m*_. A maximum of five candidates *P*_*t*_ are identified per intensity line.

**Fig. 7:**
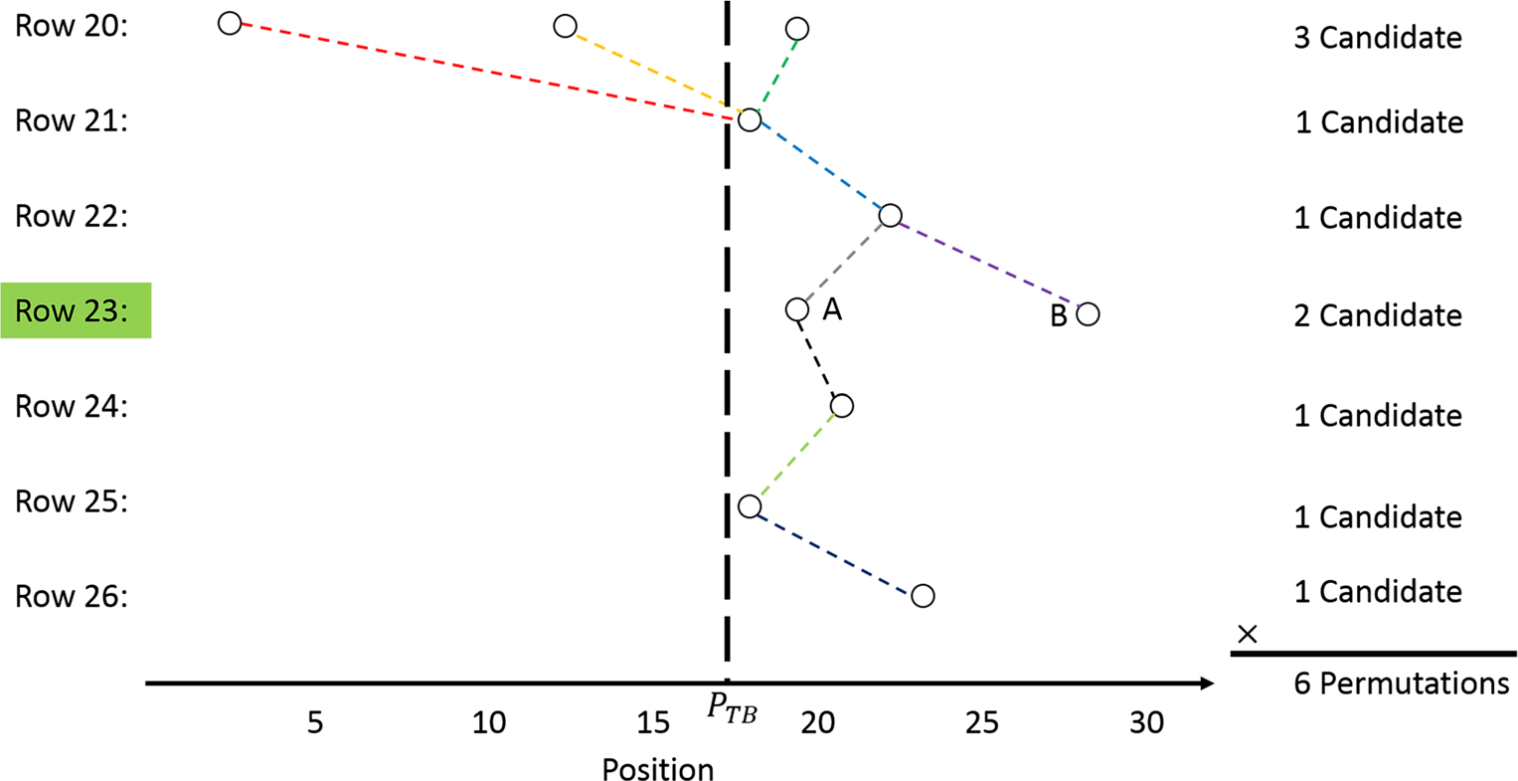
Illustration of trabecular boundary determination of row 23 (line connection between candidates (different color means different line connection), and black-dashed line represents the position of *P*_*TB*_)

**Fig. 8:**
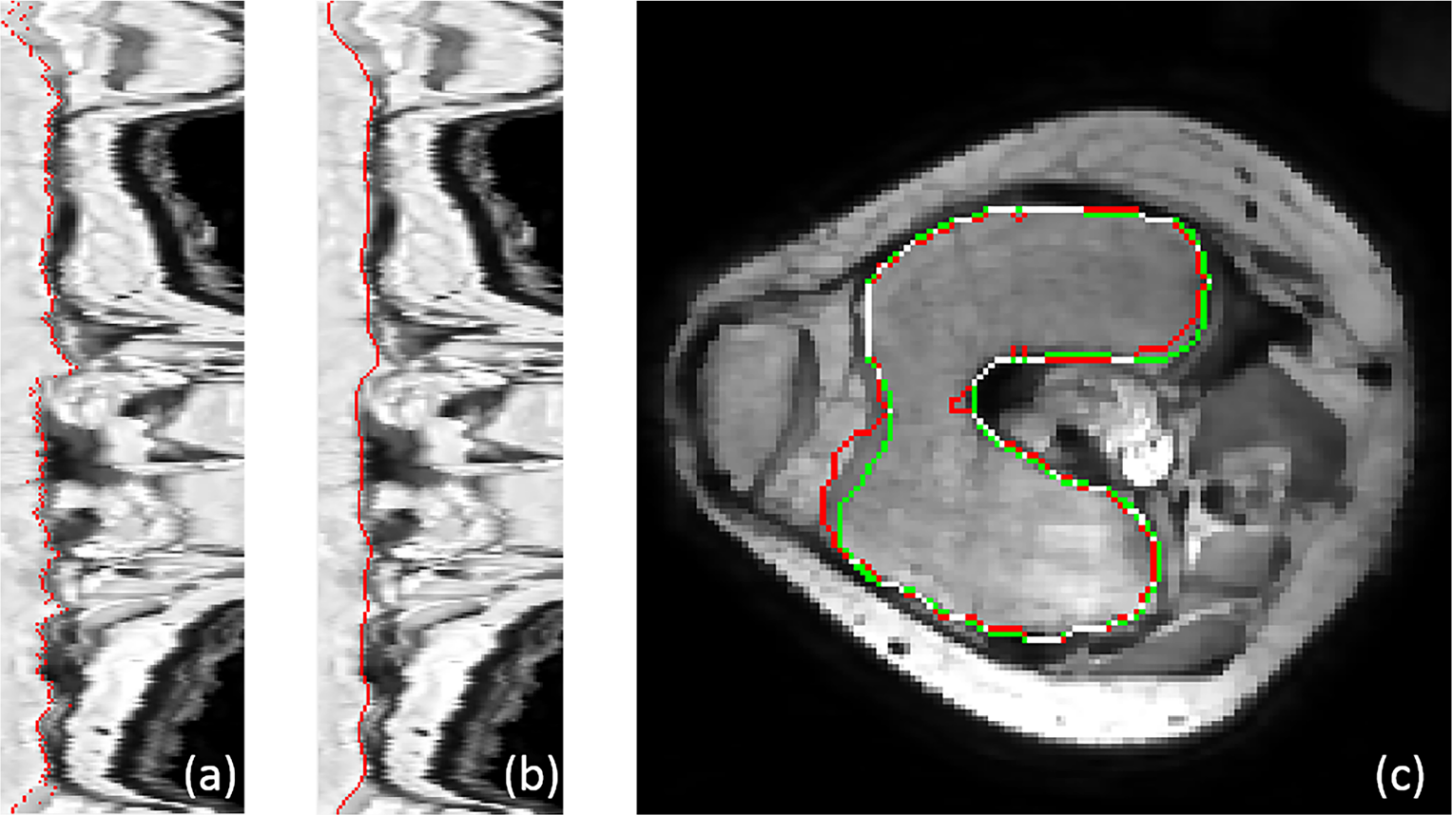
**a** Selection of trabecular boundary in IL2DI (red points). **b** Smooth version of (**a**) (red points). **c** Trabecular boundary in transverse view (red, rough trabecular boundary; green, optimized trabecular boundary; white, overlap of green and red)

To calculate the precise position of the trabecular boundary, many subsets of boundary candidates are constructed from a set of neighboring intensity lines (*M* = 7 in this study). In order to determine the suitable edge point (A or B) for the example of row 23, six permutations are obtained as shown in Fig. 7. The trabecular bone boundary is now determined as the candidates with minimal variance and closest to the rough trabecular boundary as the minimum of the cost function:

13 $$ \underset{n=1:N}{\min}\left\{{f}_{ST D}\frac{ST{D}_n}{\underset{n=1:N}{\max } ST{D}_n}+{f}_{DD}\frac{\sum_{m=1}^M\mathrm{abs}\left({P}_n^m-{P}_{TB}\right)}{\underset{n=1:N}{\max }{\sum}_{m=1}^M\mathrm{abs}\left({P}_n^m-{P}_{TB}\right)}\right\} $$

where *f*_*STD*_ and *f*_*DD*_ are the weight for standard deviation and distance deviation from the initial trabecular boundary, respectively, and defined as *f*_*STD*_ = *f*_*DD*_ = 1. *STD*_*n*_ is the standard deviation of the given permutation of trabecular candidates. ${P}_n^m$ represents *m* row of *n* permutation and *P*_*TB*_ means the position of rough trabecular boundary. The first term minimizes the distance between candidates among rows, while the second term minimizes the distance between boundary end result and initial 3DLICLS result. The boundary selection of example in Fig. 7 is *A*, and the example of selected candidates in IL2DL is shown with red points in Fig. 8a.

Then, as Fig. 8b shows, the found trabecular contour is smoothened in the IL2DI view with a Gaussian filter of kernel size 3. The found contours are then mapped back from IL2DI to transverse view and smoothened in slice direction to ensure a smooth continuous trabecular mask, as Fig. 8c shows.

### Cortical bone boundary detection

The main obstacle to extract the robust cortical edge exists in the weak contrast between cortical bone and ligament tissue. Before solving the obstacle, we make two assumptions:

1. The thickness of cortical bone on femur decreases in inferior direction, while the one on tibia decreases in superior direction [7].
2. From perspective of manual segmentation, the weak boundary is identified based on surrounding tissue among adjacent slices (the assumption is based on discussion with two experts who have segmented over 50 data sets at the orthopaedic lab for the purpose of generating FE models).

According to the assumptions, we propose two steps to solve the challenge of cortical bone determination, especially in the region with a weak edge.

Step 1 *Construction of initial cortical boundary*

**Fig. 9:**
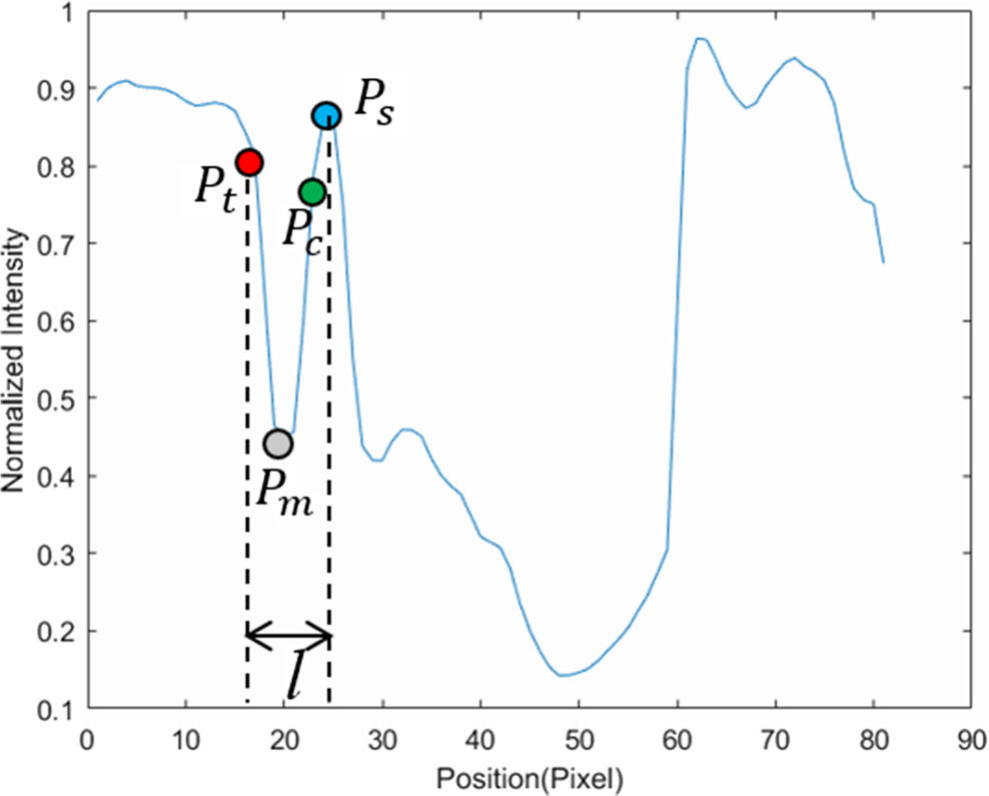
Intensity line of blue-dashed line in Fig. 7b (*P*_*t*_ represents the trabecular boundary, *P*_*m*_ represents the point with local minimum intensity, *P*_*c*_ represents the cortical boundary, *P*_*s*_ represents the maximum search range and *l* represents the distance between *P*_*t*_ and *P*_*s*_)

An initial cortical boundary is obtained by searching for the point of maximum incline after the first minimum *P*_*m*_, starting from the trabecular bone boundary *P*_*t*_ in the IDL2L (see Fig. 9).

**Fig. 10:**
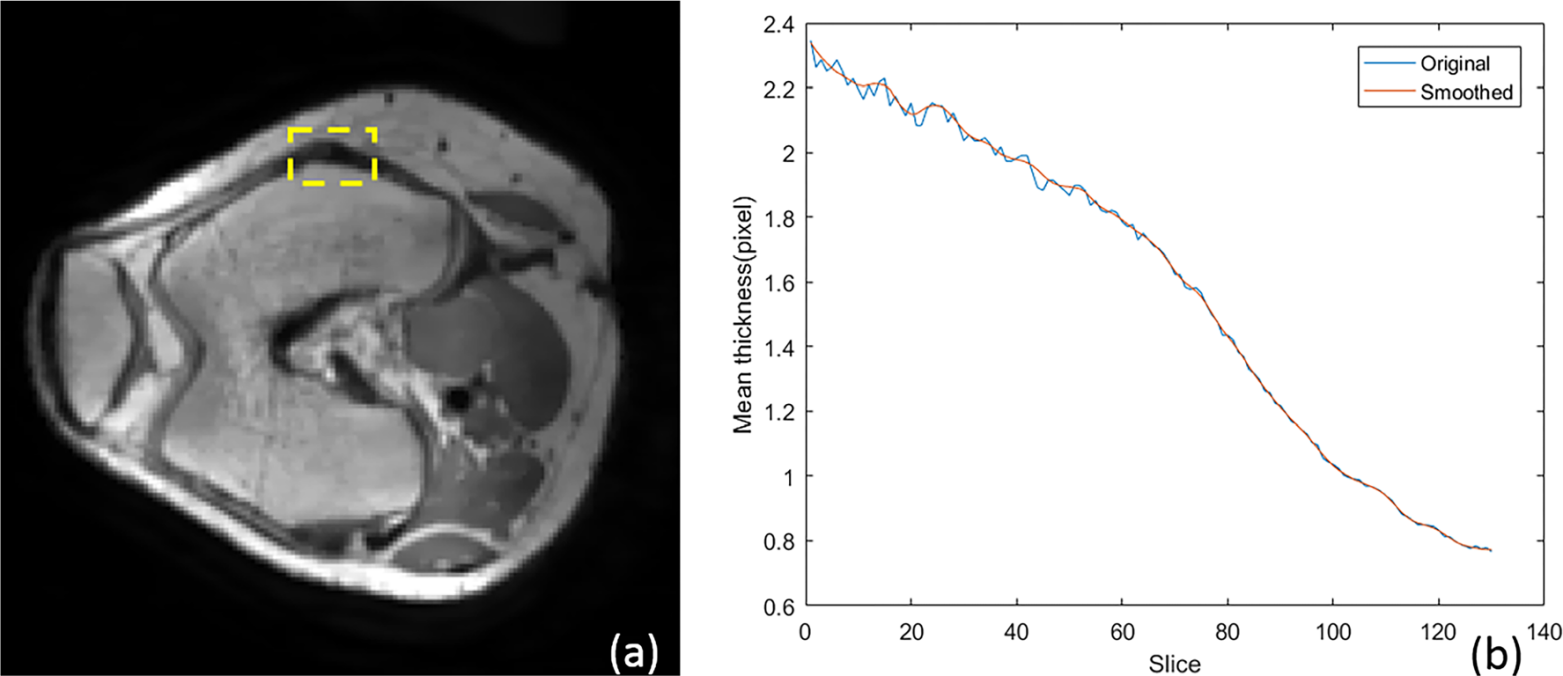
**a** Transverse view of femur (yellow box represents the connected area between cortical bone and ligament). **b** Mean cortical thickness variation along inferior direction of femur (blue, original version; red, smoothed version)

This procedure provides a first guess of the cortical bone. However, there can still be outliers in the area with noise and weak contrast, especially near the ligaments where a ligament boundary can be mistakenly selected for cortical bone (yellow box in Fig. 10a). For that matter, the actual cortical boundary is iteratively detected based on assumption 2.

Step 2 *Iterative optimization of cortical boundary*

Firstly, the average thickness *R*_*MeanC*_ along the inferior direction of femur and superior one of tibia in each slice can be calculated by using the cortical area divided by the mean perimeter, *C*_*TC*_ (average perimeter of cortical and trabecular boundaries):

14 $$ {R}_{MeanC}={A}_{Cortical}/{C}_{TC} $$

*A*_*Cortical*_ means the area of cortical bone, which calculated by the area of total bone (cortical and trabecular) and trabecular bone (*A*_*Cortical*_ = *A*_*Total*_ − *A*_*Trabecular*_). Figure 10b shows the variation of the mean cortical thickness in each slice from inferior direction for the femur and the smoothened version.

Similar to trabecular optimization, the cortical boundary is determined as the minimum of the cost function, which consists of the candidates with minimal variance and closest to position of *P*_*Cmean*_ = *P*_*TB*_ + *R*_*MeanC*_:

15 $$ \underset{n=1:N}{\min}\left\{{f}_{ST D}\frac{ST{D}_n}{\underset{n=1:N}{\max } ST{D}_n}+{f}_{DD}\frac{\sum_{m=1}^M\mathrm{abs}\left({P}_n^m-{P}_{Cmean}\right)}{\underset{n=1:N}{\max }{\sum}_{m=1}^M\mathrm{abs}\left({P}_n^m-{P}_{Cmean}\right)}\right\} $$

where *f*_*STD*_ and *f*_*DD*_ are the weight for standard deviation and distance deviation from *P*_*Cmean*_, respectively, and defined as *f*_*STD*_ = *f*_*DD*_ = 1. *STD*_*n*_ is the standard deviation of the given permutation of cortical candidates. ${P}_n^m$ represents *m* row of *n* permutation. Normally, the candidates of cortical boundary are at most three maximum incline after the trabecular boundary. Nevertheless, if the first incline is larger than the mean thickness of correspondent points of its last three layers, the position *P*_*Cmean*_is added to the candidate set of cortical boundary. To be more exact, this step simulates the assumption 2 and provides an extra option, position of *P*_*Cmean*_, in the area may exist the weak edge. The optimization of cortical boundary is an iterative procedure, which will be updated until convergence of the change of mean cortical thickness. The criterion for convergence in this study was defined as a change in mean cortical thickness between two iterations to be less than 1 pixel.

At last, same as trabecular optimization, the found cortical contour is smoothened and mapped back to transverse view.

### Evaluation

All datasets were analyzed in MATLAB 2015b. Data analysis was carried out on a conventional laptop with CPU Intel Core I7-4700MQ (2.40 GHz) and 16 GB RAM. The manual segmentation was defined as ground truth for scoring of the automatic segmentation. The manual segmentation was performed in the Mimics software environment. The outcomes were quantified with the Dice sensitivity coefficient (DSC) [5, 6, 8, 19] and the average surface distance (ASD) [5, 16].

## Results

### Comparison between 3DLICLS and 2DLICLS

**Fig. 11:**
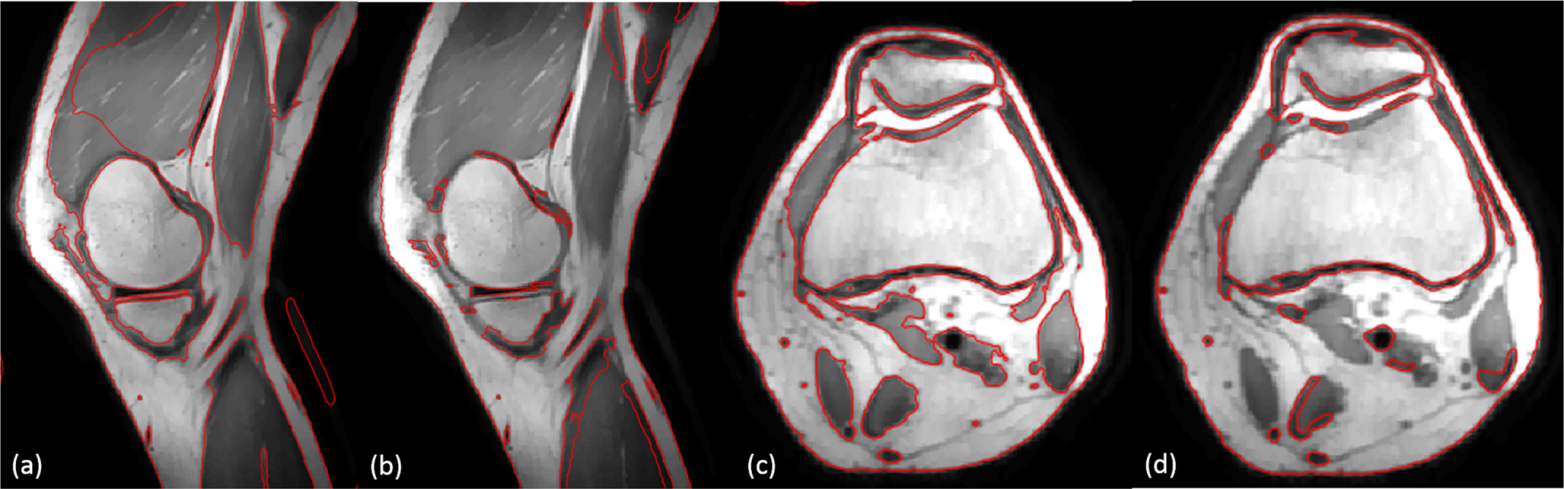
Comparison between 3DLICLS and 2DLICLS. Zero level set indicated by red contour. **a**, **c** Sagittal and axial result of 3DLICLS. **b**, **d** The sagittal and axial result of 2DLICLS

Figure 11 shows the initial trabecular result before the erosion operation between the 3DLICLS and 2DLICLS (performed the algorithm slice by slice). The result from 2DLICLS shows more leakage areas than the 3DLICLS.

### Segmentation results for trabecular bone

**Fig. 12:**
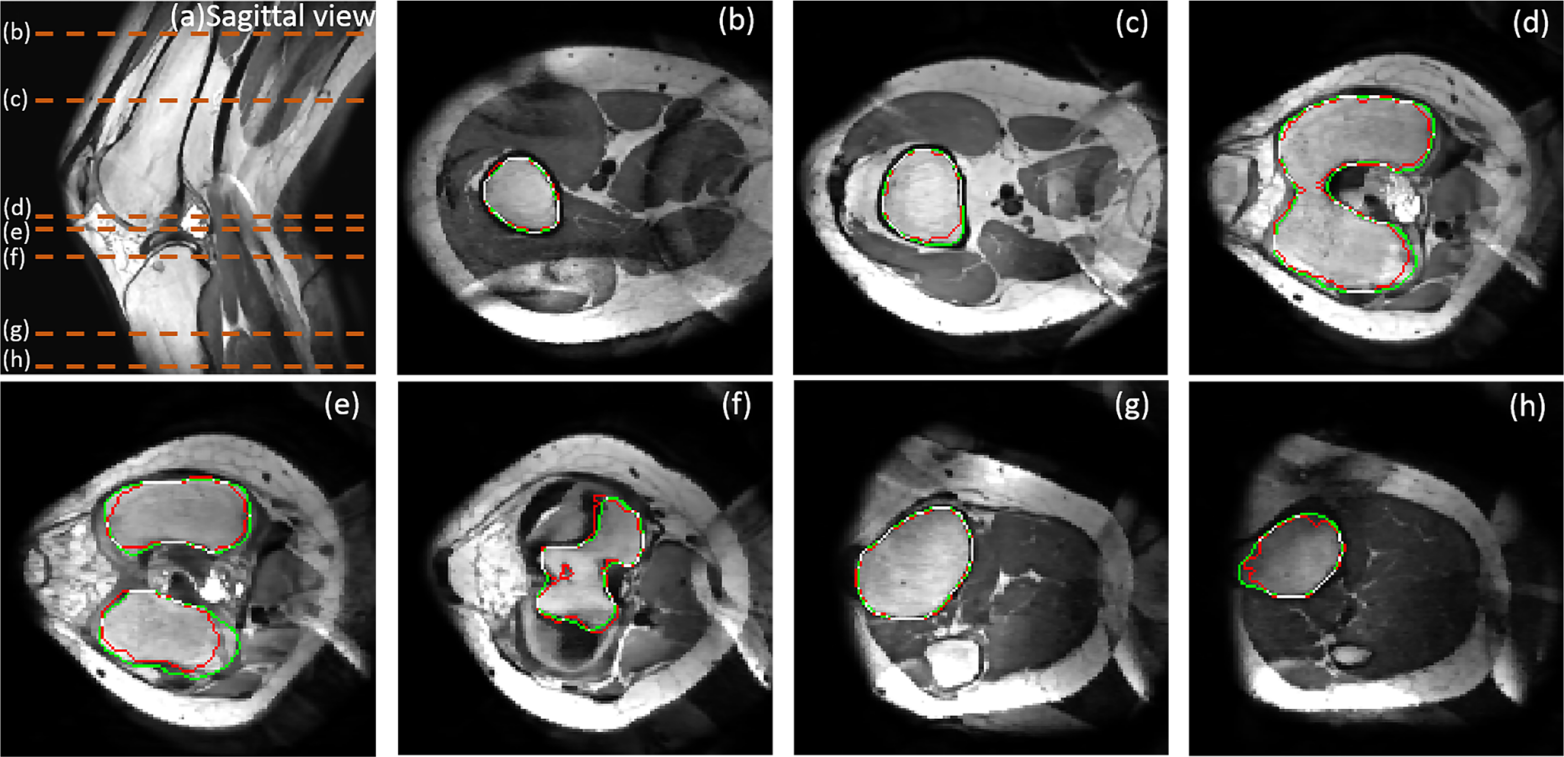
**a** Sagittal view from the result of 3DLICLS. **b**–**h** Transverse view of rough trabecular masks (red contour) and the optimized trabecular masks (green contour). White point means the overlap between rough trabecular mask and optimized mask

Figure 12 shows the final trabecular result for the first data sets in sagittal view at mid-slice position and in transversal view at several key positions. The red contour represents rough trabecular result after 3DLICLS and image morphological operation and the green contour is the trabecular result after optimization in 2.5.

Generally, femur and tibia are isolated with 3DLICLS, as Fig. 12b–h shows. The inhomogeneity problem increases near the outer slices of the FOV as can be seen in Fig. 12h. 3DLICLS however determines the bias field and is still able to segment the trabecular bone robustly in this area.

### Segmentation results for cortical bone

**Fig. 13:**
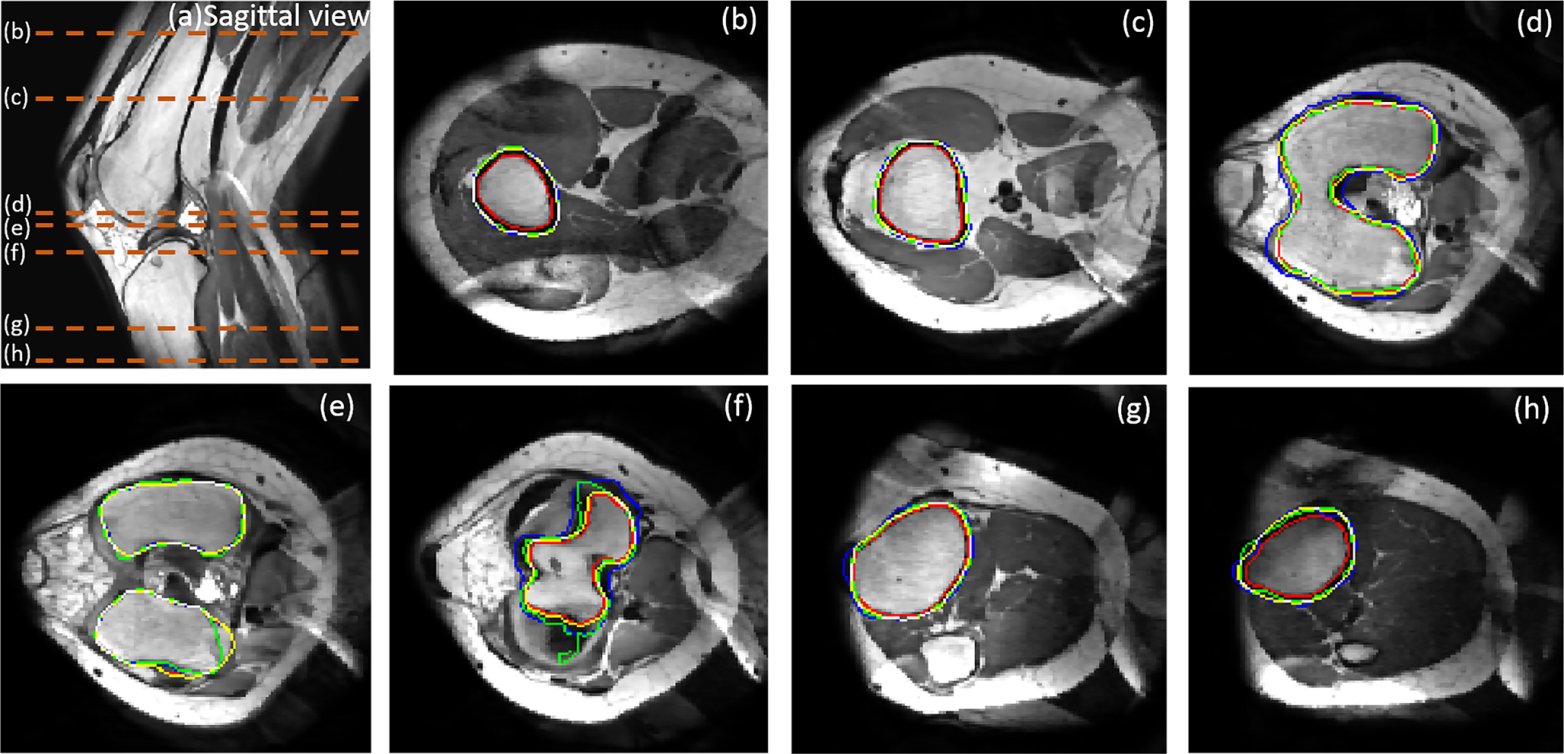
**a** Sagittal view to show the position of (b)–(h). **b**–**h** Comparison results from transverse view. Green contour is manual segmentation, yellow contour is cortical boundary of proposed method, blue contour is the initial cortical mask (2.6 step (1)), red contour is the optimized trabecular mask and white point means the overlap between proposed method and manual segmentation

Figure 13 shows the cortical segmentation results including cortical bone guess (red) in first maximum incline (2.6 step (1)), final cortical mask within proposed method (yellow) and manual segmented mask (green). Plus, white point represents the overlap between proposed method and manual segmentation.

From (b) to (e) and (h) to (f), the phenomenon of cortical bone thinning towards the femur condyles and top of tibia can be noticed respectively, where the red contour (trabecular boundary) moves more and more towards the yellow and green contour (automatic and manual segmentation of the cortical boundary).

In the shaft area without inhomogeneity (regions (b) and (c)), there is virtually full agreement between manual segmentation and our method. Difficulties arise in the areas containing transition from cortical bone to cartilage and/or ligament, depicted in (d)–(g). Despite the weak edges between ligament and cortical bone, the automatic segmentation still displays minimal disagreement with the manual one. Furthermore, the performance in the region with inhomogeneity (h) also displays convincing result.

### Convergence of cortical boundary detection

**Fig. 14:**
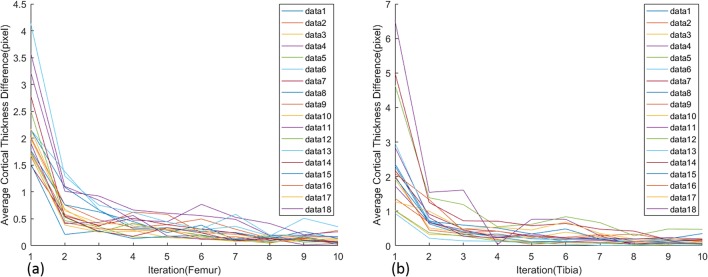
Average cortical thickness difference between neighbor iterations. **a** Femur. **b** Tibia

Figure 14a, b shows the difference of sum of average cortical thickness along the iteration for femur and tibia among the 18 data, respectively. Generally, the change becomes converged after eight iterations, but the result normally changes little after four iterations.

### Segmentation accuracy

**Fig. 15:**
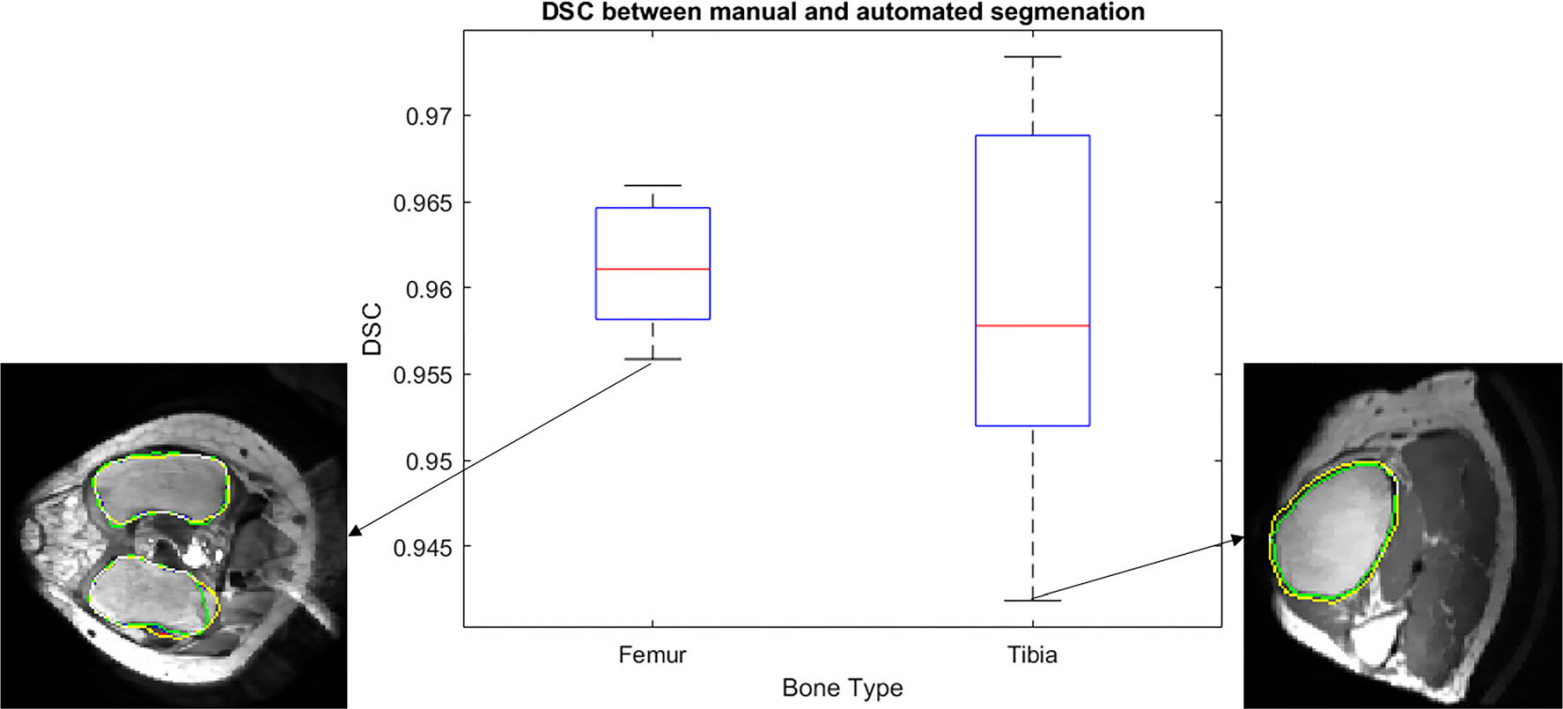
Box plot of comparison between automatic and manual segmentation calculated in DSC scores (green contour is cortical boundary of manual segmentation, yellow contour is cortical boundary of proposed method, white point represents the overlap between green and yellow point)

**Fig. 16:**
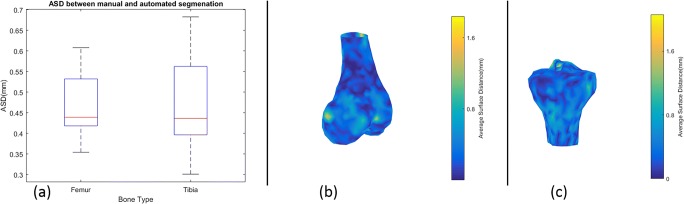
**a** Box plot of average surface distance difference between automatic and manual segmentation for femur and tibia. **b**, **c** Distance difference in 3D view for femur and tibia, respectively

As the boxplots in Fig. 15 show, the average DSC are 0.9611 ± 0.0052 for the femur and 0.9591 ± 0.0173 for the tibia. Two typical situations with low DSC score are also shown in Fig. 15. The average distances to surface between the automatically and manually segmented bones, 0.4649 ± 0.1430 mm for the femur and 0.4712 ± 0.2113 mm for tibia, are shown in Fig. 16a, and a 3D difference for femur and tibia is shown schematically in Fig. 16b, c.

### Segmentation time

The average time needed to segment one dataset (femur and tibia) with a matrix of 336 × 336 × 222 voxels was around 250 s and 2.5 h for automatic and manual segmentation, respectively. Hence, the prosed method is efficient and promising for assisting segmentation research.

## Discussion

In this study, we proposed an automatic workflow to segment the cortical and trabecular bone of femur and tibia in proton density weighted MRI. A 3D level set-based algorithm is used to segment the rough trabecular boundary and remove any slow varying inhomogeneity. Trabecular and cortical bone boundaries are detected from the intensity profiles along normal vectors generated from the trabecular surface. Upon testing of the method in 18 datasets, the algorithm demonstrated its capability to handle field inhomogeneity and correctly detect trabecular and cortical bone over the full field of view including weak edges near ligament and thinning cortical bone. Scoring of the proposed segmentation method using manual segmentations as a reference yielded DSC over 95% and ASD errors less than 0.5 mm for both femur and tibia.

Table 1 shows a comparison of evaluation results from similar studies in recent literatures. Although our method’s results are well within the range of success rates as reported from literatures, we must stress the difficulty in direct comparison between methods because of the differences in workflow. Shan et al. [19] and Fripp et al. [6] for instance use prior data, whereas we do not. Guo et al. [8] reported scores on trabecular bone segmentation only, and Pang et al. [16] reported average surface distances for specific slice locations versus over the whole bone surface.

**Table 1:** Result comparison between proposed method and previous studies

	DSC		ASD (mm)	
	Femur	Tibia	Femur	Tibia
Dodin et al [5]	0.94 ± 0.05	0.92 ± 0.07	0.50 ± 0.12	0.37 ± 0.09
Pang et al. [16]	–	–	0.459 ± 0.187	0.845 ± 0.392
Shan et al. [19]	0.970 ± 0.011	0.967 ± 0.012	–	–
Guo et al. [8]	0.94	0.94	–	–
Fripp et al. [6]	0.96	0.96	–	–
This study	0.9611 ± 0.0052	0.9591 ± 0.0173	0.4649 ± 0.1430	0.4712 ± 0.2113

There are several limitations in this study. Firstly, manual segmentations from a trained expert were used as a ground truth for scoring the automatic method. As the exact boundary between cortical bone and ligament is often not completely clear even for orthopaedic surgeons, this ground truth is subject to debate. Hence, the results presented in this study only show the method’s capability to simulate the manual evaluation of cortical bone. Secondly, the patients whose data was used in this study were all in relatively good health as the knee is concerned. Patients with pathologies that affect the bone and cartilage (e.g. osteoporosis, osteoarthritis, bone marrow lesions) may require re-tuning of the parameters of the automatic segmentation algorithm. This requires bigger datasets and clinical applications.

This fast and robust segmentation of trabecular and cortical bone boundary of the femur and tibia has the potential of providing a basis for surgical planning and more accurate finite element models of the knee joint. By removing the large workload that is involved in manual segmentation of MRI images, these methods can potentially be introduced in the clinic and in large-scale research projects. In this study, proton density weighted contrast was used, because of its wide availability, short scan time and orthopaedic relevance. The method, however, could also be adjusted to extract bone from other types of contrast, provided there is an overall difference in contrast between trabecular bone, cortical bone and adjacent tissues, and there is enough consistency in the trabecular to cortical bone boundary to correct any weak edges using its surroundings.

Furthermore, as the method contains no substantial assumptions, constraints or premises with respect to the shape of the bone but rather to the contrast, it is feasible to extend this method to the shoulder and elbow joint.

## Conclusion

In this paper, we presented and evaluated an automatic workflow to segment the trabecular and cortical bone of femur and tibia with PDW sequence in MRI. Initial results compared with manual segmentation indicate the possibility to provide an automatic segmentation to researchers and clinical doctors to perform further analysis rather than the time-consuming manual segmentation.

Future studies will include the following: an increase in the number of patients of the test group; an extension of the method to determine the knee bone and cartilage; and an automated workflow to provide clinically relevant parameters, such as tibia tubercle-trochlear groove distance (TT-TG) and patella tilt.
